# Is there an association between gender stereotypes and sexual risk attitudes and behaviors? A population-based study among Peruvian adolescents

**DOI:** 10.1093/inthealth/ihad120

**Published:** 2024-01-09

**Authors:** Diana Manuela Ticona, Ariana Gabriela Musaja-Cruz, Paula Regina Durand-Anahua, Raul Eduardo Escobar-Cabezas, Luz Mirian Mamani, Crislee Elizabeth Lopez

**Affiliations:** Hospital Nacional Docente Madre Niño San Bartolomé, Oficina Ejecutiva de Planeamiento Estratégico, 15001, Lima, Peru; Universidad Nacional Mayor de San Marcos, Facultad de Medicina, 15001, Lima, Peru; Universidad Privada de Tacna, Centro de Investigación de Estudiantes de Medicina (CIESMED), 23003, Tacna, Peru; Universidad Privada de Tacna, Centro de Investigación de Estudiantes de Medicina (CIESMED), 23003, Tacna, Peru; Universidad Privada de Tacna, Centro de Investigación de Estudiantes de Medicina (CIESMED), 23003, Tacna, Peru; Universidad Privada de Tacna, Centro de Investigación de Estudiantes de Medicina (CIESMED), 23003, Tacna, Peru; Hospital Regional Honorio Delgado Espinoza, Oficina de epidemiología y salud ambiental, 04001, Arequipa, Peru

**Keywords:** adolescent, reproductive health, sexism, sexual behavior, sexual health

## Abstract

**Background:**

Adolescents, particularly those aged 14 to 15 y, often begin exploring their sexuality, during which time they are more vulnerable to traditional influences and ideologies imposed by society. This study aimed to identify the association between more traditional attitudes toward women and sexual risk attitudes and behaviors in Peruvian adolescents.

**Materials and methods:**

Cross-sectional study with data from the fifth round of the Young Lives study with 1860 adolescents aged 14 and 15 y. Population characteristics were described by relative frequencies and using chi-squared test with p-value. The 12 items of the Attitudes toward Women Scale for Adolescents instrument were analyzed by relative frequencies and mean with standard deviation. For further analysis, the values of the global scores of all participants were divided into two categories, using the median as the cutoff point, where the group with higher scores indicated that these adolescents had more traditional attitudes (or more gender stereotypes). More traditional attitudes were associated with sexual risk attitudes and behaviors, with crude prevalence ratio (PR) and then adjusted prevalence ratio (aPR) with 95% CI. The Universidad Privada de Tacna’s ethics committee approved the research protocol.

**Results:**

Adolescents with more traditional attitudes were more likely to have sexual risk attitudes compared to those with less traditional attitudes. In addition, adolescents with more traditional attitudes were 2.6 times more likely to have at least one sexual intercourse while drunk as compared to the reference group (95% CI: 1.43–4.74; p=0.002).

**Conclusions:**

The expression of more traditional attitudes toward women was associated with sexual risk attitudes. However, there was no association with most of the sexual risk behaviors studied, except for the higher probability of having at least one sexual intercourse while drunk.

## Introduction

Gender stereotypes, also called gender roles or sexism, encompass a set of ideas or prejudices about the attributes, characteristics and social roles that both men and women should possess or perform.^1^ Their influence during adolescence, an age when sexuality become important, can act as a determining factor in learning and practicing sexual activities.^2^ Adolescents are more vulnerable to negative sexual outcomes compared to other age groups due to their susceptibility and influence, and exposure to various sexual and reproductive health risk factors, including feeling pressured to engage in sexual activity, unsafe sex practices, sexually transmitted diseases (STDs) or unwanted pregnancies.^3,4^ Furthermore, according to the WHO, men enjoy social and institutional privileges over women with greater decision-making power in their sexual relationships, especially in middle- and low-income countries.^5^

Sexual risk behaviors such as not using condoms or sexual risk attitudes such as thinking that women cannot get pregnant during their first sexual intercourse influence the sexual health of adolescents. In addition, gender inequality is a key factor in the decision making process at the time of initiating a sexual relationship.^6^ Thus, in a sample of Spanish adolescents an association was identified between high gender stereotype and inconsistent condom use in males, as well as a greater number of sexual partners in females who showed a high degree of sexism.^7^ Likewise, a study in rural South Africa showed that those who had not used condoms in the previous year were significantly more conservative than consistent users.^8^ However, Perrote et al. found that Latino males in the United States with high gender stereotypes were more likely to use condoms in their sexual relationships.^9^ A new theory explains the existence of a conservative but less violent and sexually risky male position, that is men with traditional attitudes toward women but responsible in their sexual lives.^10^ Although current evidence indicates an association between gender stereotypes and risky sexual practices, it is important to recognize that each country has its own idiosyncrasy and different realities.

Adolescence is a critical transitional stage between childhood and adulthood, during which adolescents could initiate sexual activity at an early age and without adequate use of contraceptive methods; exposing themselves to risks that can compromise their sexual health, psychological well-being and social development^11^. The number of adolescents who initiate sexual activity before the age of 15 has increased in Peru; it is at this age, specifically between 14 and 15 years old, that adolescents begin to explore their sexuality with the consequent formation of their sexual attitude and behavior, making them more vulnerable to social influences and traditional ideologies.^12^ Furthermore, in the search for the acceptance of their peers they tend to ignore the risks caused by their inexperience and lack of knowledge.^13^ Therefore, this study aimed to identify the association between more traditional attitudes toward women and sexual risk attitudes and behaviors in Peruvian adolescents.

## Materials and Methods

### Study design, space and time

Cross-sectional analysis using information from the database of the Young Lives study, an ongoing prospective cohort about the changing nature of child poverty in four low-income countries (Ethiopia, Andhra Pradesh-India, Vietnam and Peru).

### Population, sample, sampling and procedures

This research used data from the fifth round of the Young Lives study conducted during 2016, where adolescents were aged 14 and 15 y.

In Peru, sentinel communities were chosen using a multistage stratified cluster random sampling strategy. The sentinel sites were districts, selecting 20 sentinel communities from 1818 available districts. Of the total selected, 5% of the richest districts were not included in order to sample the poor areas as expected, resulting in a final 75% declared as poor. All field workers and supervisors were men and women with the minimum educational requirements, previous experience in data collection, and fluent in speaking and writing the languages of the localities in which they were assigned. Through an interview with the mother and the adolescent, the data were recorded in questionnaires, which were entered into a database available at the following link https://www.younglives.org.uk/. Consequently, during the fifth round, the research team surveyed 1860 adolescents (938 males and 922 females). Any additional methodological details can be found in the report on the design and methods of the Young Lives study in Peru.^14^ For the purposes of this analysis, participants with incomplete data on any of the variables studied were excluded. Likewise, the manuscript was written following the guidelines of Strengthening the Reporting of Observational Studies in Epidemiology.^15^

### Definition of variables and measurement method

#### Gender stereotypes

The level of adolescent gender stereotyping was measured using the Attitudes toward Women Scale for Adolescents (AWSA). This instrument consists of 12 items (V01-V12), each item representing an attitude to which participants respond according to a Likert-type scale ranging from 1 (‘strongly agree’) to 4 (‘strongly disagree’). Items V03, V05, V07, V09 and V12 were reverse-scored. All scores were then summed and divided by 12 to maintain the item metric, producing a final individual score (range between 1 and 4). For further analysis, the values of the final individual scores of all participants were divided into two categories, using the median as the cutoff point, where the group with higher scores indicated that these adolescents had less traditional attitudes (or less gender stereotypes). The instrument was validated in Spanish in a sample of Ecuadorian and Bolivian adolescents, with a Cronbach's alpha of 0.61.^16^

#### Sexual risk attitudes

Adolescents' sexual risk attitudes were assessed using the statements: ‘a girl cannot get pregnant the first time she has sex’; ‘if a girl washes herself after sex, she will not get pregnant’; ‘using a condom can prevent you from getting a disease through sex’; ‘a person who looks very healthy cannot pass on a disease through sex’; and ‘a person can get HIV or AIDS by having sex’. Participants responded to each statement with true, false or I don't know. Statements 1, 2 and 4 that were answered with I don't know or true were considered as sexual risk attitudes; while statements 3 and 5 with don't know or false answers were assessed as sexual risk attitudes.

#### Sexual risk behaviors

The sexual risk behaviors of sexually active adolescents were analyzed through the questions: ‘How old were you when you had sex for the first time?’; ‘How many partners have you ever had intercourse with?’; ‘The first time you had sex, what did you do to prevent getting pregnant or getting your partner pregnant?’; ‘Nowadays, what do you do to prevent getting pregnant or getting your partner pregnant?’; ‘Last time you had sex, what did you do to prevent getting pregnant or getting your partner pregnant’; and ‘Have you ever been drunk from alcohol while having sex?’. Early sexual debut was defined as having sex for the first time at or before age 14 for question 1; multiple sexual partners as having more than one sexual partner for question 2; inconsistent condom use as having no condom use for at least one of questions 3, 4 or 5; no condom use as having no condom use for all questions 3, 4 and 5; and sexual intercourse while drunk when the participant answered yes for question 6.

#### Sociodemographic characteristics

Other variables were sex (woman or man), age (14 or 15 y), area of residence (rural or urban), socioeconomic status (top, middle or bottom wealth tercile), household size (5 to less or more than 5), mother's educational level (less than 7 y or 7 y or more), father's educational level (less than 7 y or 7 y or more), and school attendance (yes or no).

### Statistical analysis

Selected files of interest were exported to RStudio 4.2.0 for analysis.

The sociodemographic characteristics, sexual risk attitudes and behaviors of the study population were described by calculating relative frequencies and their respective 95% CIs, comparing the groups of less and more traditional attitudes. Likewise, the responses to the 12 items of the AWSA instrument were described by calculating relative frequencies and measures of central tendency and dispersion as mean and standard deviation (SD), of the study population and comparing according to sex of the participants. Chi-square for categorical variables and Student's t-test for quantitative variables with their respective p-values were used for group comparisons.

To evaluate the magnitude of associations between more traditional attitudes with sexual risk attitudes and more traditional attitudes with sexual risk behaviors, Poisson regression models with robust variance were performed to estimate prevalence ratios (PRs) with their 95% CIs. A crude model was constructed reporting the crude PRs, and all sociodemographic variables that obtained a statistically significant p-value (p<0.20) were then included in the adjusted model to report adjusted prevalence ratios (aPRs).

### Ethical considerations

The Young Lives study received approval from the University of Oxford ethics committees prior to pilot testing and the first round of data collection. In Peru, the Research Ethics Committee of the Institute of Nutritional Research approved the study. Written parental informed consent as well as verbal adolescent assent was obtained in each round. Likewise, the Universidad Privada de Tacna’s ethics committee approved the research protocol (identification code: 055-FACSA-UI, 16 April 2021).

## Results

### Characteristics of the study population

**Figure 1.**
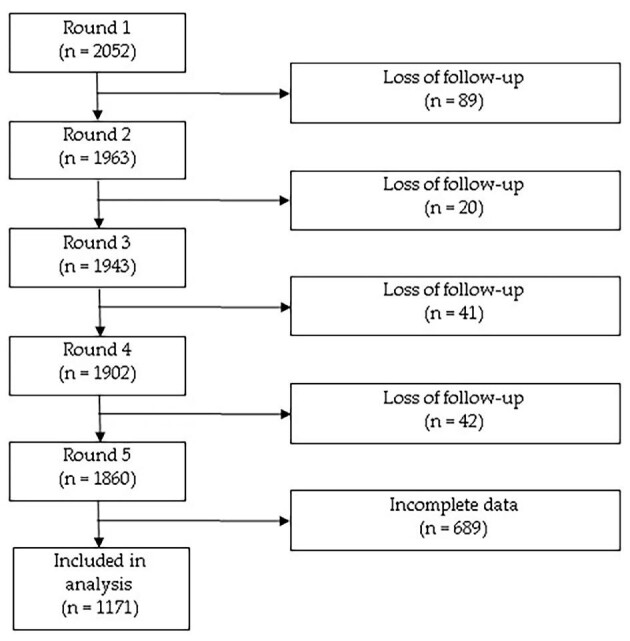
Flowchart of the participants of the Peruvian younger cohort in the Young Lives study.

In the fifth round, 1860 adolescents were evaluated; of these, 689 were excluded due to lack of information on any of the variables, so data from 1171 adolescents were analyzed (Figure 1). The mean age was 14.45 (SD 0.50) years where 51.8% were men and 98.4% attended school; furthermore, this population was predominantly urban, in the top wealth tercile, with 5 or less members in the household, with 7 or more years of parental education. A total of 16.8% (95% CI: 14.7–19.1) of the total adolescents showed more traditional attitudes. Likewise, 61.9% of the total participants with more traditional attitudes were men (p=0.002). Information on the characteristics of the population according to less or more traditional attitudes is detailed in Table 1.

**Table 1.** Description of the sociodemographic characteristics, sexual risk attitudes and behaviors according to less or more traditional attitudes of the study population

	Total	Less traditional attitudes	More traditional attitudes	
Sociodemographic characteristics	n=1171 % (95% CI)	n=974 % (95% CI)	n=197 % (95% CI)	p-Value*
Age				
14 y	54.6 (51.7–57.5)	54.5 (51.3–57.7)	54.8 (47.6–61.9)	0.938
15 y	45.4 (42.6–48.3)	45.5 (42.3–48.7)	45.2 (38.1–52.4)	
Sex				
Woman	48.2 (45.3–51.1)	50.2 (47.0–53.4)	38.1 (31.3–45.2)	0.002
Man	51.8 (48.9–54.7)	49.8 (46.6–53.0)	61.9 (54.8–68.7)	
Area of residence				
Urban	75.4 (72.877.9)	78.0 (75.3–80.6)	62.4 (55.3–69.2)	<0.001
Rural	24.6 (22.2–27.2)	22.0 (19.4–24.7)	37.6 (30.8–44.7)	
Socioeconomic status				
Top wealth tercile	35.1 (32.4–37.9)	38.1 (35.0–41.2)	20.3 (14.9–26.6)	<0.001
Middle wealth tercile	33.6 (30.9–36.4)	34.3 (31.3–37.4)	30.5 (24.1–37.4)	
Bottom wealth tercile	31.3 (28.6–34.0)	27.6 (24.8–30.5)	49.2 (42.1–56.4)	
Household size				
5 members or less	61.7 (58.8–64.5)	63.4 (60.2–66.4)	53.3 (46.1–60.4)	0.008
More than 5 members	38.3 (35.6–41.2)	36.6 (33.6–39.8)	46.7 (39.6–53.9)	
Mother's educational level				
7 y or more	56.5 (53.6–59.4)	59.6 (56.4–62.7)	41.6 (34.7–48.8)	<0.001
Less than 7 y	43.5 (40.6–46.4)	40.4 (37.4–43.6)	58.4 (51.2–65.3)	
Father's educational level				
7 y or more	66.4 (63.6–69.1)	69.3 (66.3–72.2)	51.8 (44.6–58.9)	<0.001
Less than 7 y	33.6 (30.9–36.4)	30.7 (27.8–33.7)	48.2 (41.1–55.4)	
School attendance				
Yes	98.4 (97.5–99.0)	98.7 (97.7–99.3)	97.0 (93.5–98.9)	0.083
No	1.6 (1.0–2.5)	1.3 (0.7–2.3)	3.0 (1.1–6.5)	
Sexual risk attitudes	n=1171 % (95% CI)	n=974 % (95% CI)	n=197 % (95% CI)	p-Value*
A girl cannot get pregnant the first time she has sex				
False	44.3 (41.5–47.2)	47.3 (44.2–50.5)	29.4 (23.2–36.3)	<0.001
True/I don't know	55.7 (52.8–58.6)	52.7 (49.5–55.8)	70.6 (63.7–76.8)	
If a girl washes herself after sex, she will not get pregnant				
False	46.8 (43.9–49.7)	49.9 (46.7–53.1)	31.5 (25.1–38.5)	<0.001
True/I don't know	53.2 (50.3–56.1)	50.1 (46.9–53.3)	68.5 (61.5–74.9)	
Using a condom can prevent you from getting a disease through sex				
False/I don't know	32.4 (29.7–35.1)	30.2 (27.3–33.2)	43.1 (36.1–50.4)	<0.001
True	67.6 (64.8–70.2)	69.8 (66.8–72.7)	56.9 (49.6–63.9)	
A person who looks very healthy cannot pass on a disease through sex				
False	45.2 (42.3–48.1)	48.1 (45.0–51.3)	30.5 (24.1–37.4)	<0.001
True/I don't know	54.8 (51.9–57.7)	51.9 (48.7–55.0)	69.5 (62.6–75.9)	
A person can get HIV or AIDS by having sex				
False/I don't know	18.7 (16.5–21.1)	15.9 (13.7–18.4)	32.5 (26.0–39.5)	<0.001
True	81.3 (78.9–83.5)	84.1 (81.6–86.3)	67.5 (60.5–74.0)	
Sexual risk behaviors	n=122 % (95% CI)	n=102 % (95% CI)	n=20 % (95% CI)	p*
Early sexual debut	66.4 (57.3–74.7)	66.7 (56.7–75.7)	65.0 (40.8–84.6)	0.885
Multiple sexual partners	52.5 (43.2–61.6)	53.9 (43.8–63.9)	45.0 (23.1–68.5)	0.465
Inconsistent condom use	51.6 (42.4–60.8)	56.9 (46.7–66.6)	25.0 (8.7–49.1)	0.009
No condom use	24.6 (17.3–33.2)	26.5 (18.2–36.1)	15.0 (3.2–37.9)	0.276
Sexual intercourse while drunk	27.9 (20.1–36.7)	22.6 (14.9–31.9)	55.0 (31.5–76.9)	0.003

*Chi-square test.

### Attitudes toward women according to the AWSA instrument

**Figure 2.**
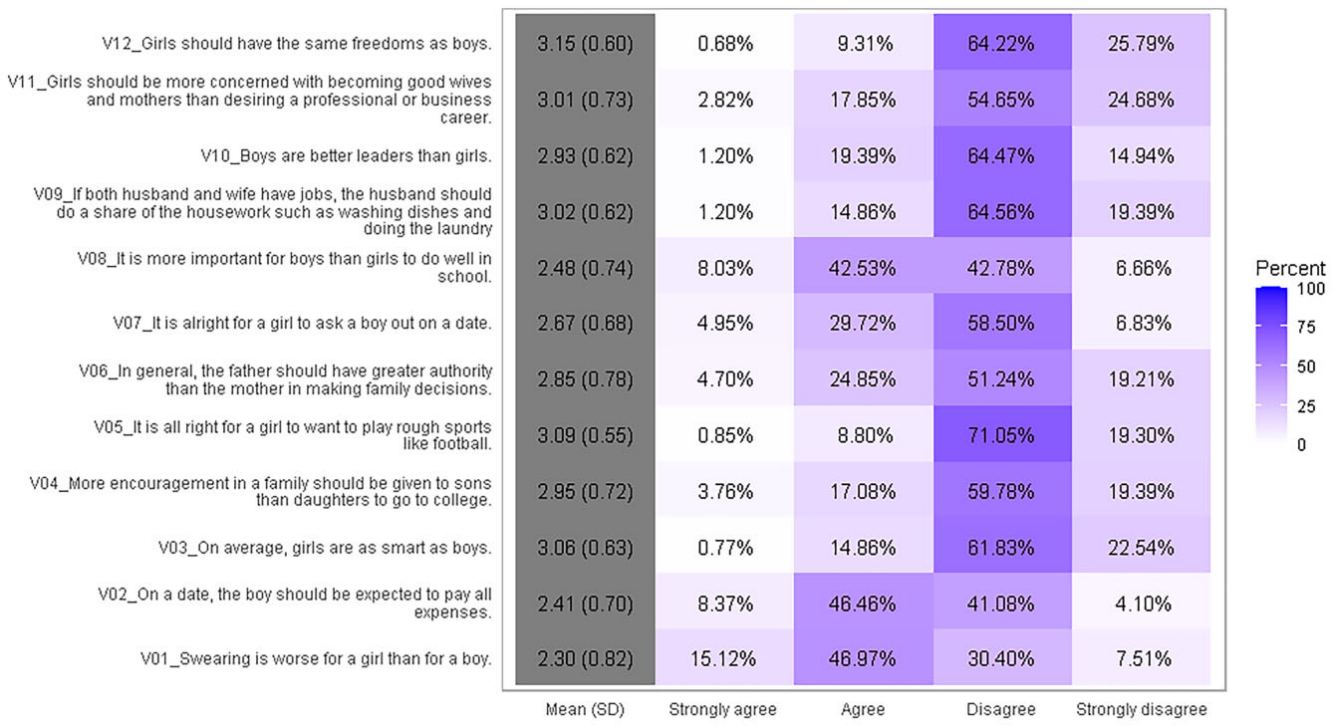
Description of answers in each item of the Attitudes toward Women Scale for Adolescents instrument of the study population.

**Figure 3.**
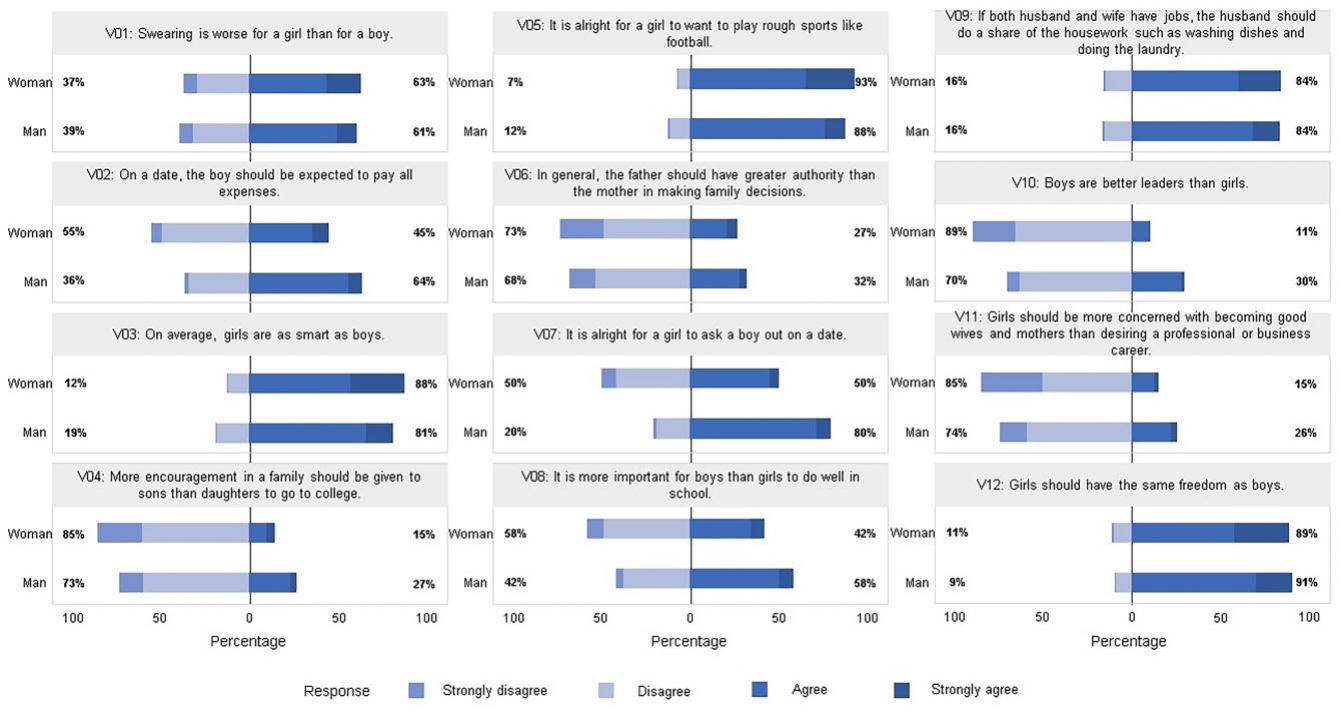
Description of responses in each item of the Attitudes toward Women Scale for Adolescents instrument, according to the sex of the study population.

Figure 2 shows the distribution of responses to each item of the AWSA instrument, where the mean total score of the participants was 2.83 (SD 0.32). Women expressed less traditional attitudes than men with mean scores of 2.90 (SD 0.33) and 2.76 (SD 0.29), respectively (p=<0.001). In all, 90.35% of total participants disagreed or strongly disagreed with the statement ‘it is all right for a girl to want to play rough sports like football’. In addition, 62.09% of all adolescents agreed or strongly agreed with the statement ‘swearing is worse for a girl than for a boy’. The distribution of responses to each item by sex of the participants is summarized in Figure 3. Responses differed significantly by sex; women disagreed or strongly disagreed more than men on items V02, V04, V06, V07, V08, V10 and V11 (p=<0.001 for all seven items). However, on items V01, V03, and V05, women agreed or strongly agreed more than men (p=<0.001 on all three items).

### Association between more traditional attitudes and sexual risk attitudes and behaviors

Less traditional attitudes were considered as the reference group in the adjusted models (Table 2). Adolescents with more traditional attitudes compared to less traditionalists were more likely to have sexual risk attitudes. However, there was no association between sexually active adolescents with more traditional attitudes and most of the sexual risk behaviors studied; only in the case of adolescents who had at least one sexual intercourse while drunk, the adjusted PR was 2.60 (more traditional vs less traditional attitudes).

**Table 2.** Association between more traditional attitudes, sexual risk attitudes and behaviors of the study population

	PR	95% CI	p-Value	PRa*	95% CI	p-Value
Sexual risk attitudes						
A girl cannot get pregnant the first time she has sex	1.34	1.20–1.49	<0.001	1.29	1.16–1.45	<0.001
If a girl washes herself after sex, she will not get pregnant	1.37	1.22–1.53	<0.001	1.33	1.18–1.49	<0.001
Using a condom can prevent you from getting a disease through sex	1.43	1.19–1.72	<0.001	1.27	1.05–1.54	0.014
A person who looks very healthy cannot pass on a disease through sex	1.34	1.20–1.50	<0.001	1.27	1.14–1.43	<0.001
A person can get HIV or AIDS by having sex	2.04	1.59–2.62	<0.001	1.60	1.24–2.07	<0.001
Sexual risk behaviors						
Early sexual debut	0.98	0.69–1.39	0.888	1.01	0.72–1.41	0.978
Multiple sexual partners	0.84	0.50–1.40	0.494	0.96	0.58–1.58	0.876
Inconsistent condom use	0.44	0.20–0.96	0.039	0.47	0.21–1.02	0.057
No condom use	0.57	0.19–1.70	0.310	0.61	0.19–1.93	0.397
Sexual intercourse while drunk	2.44	1.43–4.18	0.001	2.60	1.43–4.74	0.002

*Model adjusted for sex, area of residence, socioeconomic status, mother's educational level, father's educational level, household size and school attendance.

## Discussion

The AWSA instrument identified encouraging results regarding progress in the elimination of gender stereotypes. The findings showed that on average, attitudes toward women among Peruvian adolescents were less traditional. A result similar to that reported in the studies by De Meyer et al. in Ecuador and Bolivia, and Kemigisha et al. in Uganda, both of whom applied the same AWSA scale.^17,18^ However, this differs from findings in a study in Cuba using a different measurement instrument, which revealed a high level of gender stereotype incidence in adolescents across almost all analyzed dimensions.^19^ The Global Gender Gap Report 2023 informed that the overall progress achieved toward gender parity has increased by a total of 4.1 percentage points since 2006, and Latin America and the Caribbean region has improved the most with an increase of 8.4 percentage points. Nevertheless, upon closer examination of sub-index, Peru finds itself in the quartile with the highest gender gap in educational attainment and health/survival.^20^ Certainly, economic, political, social and cultural factors constitute the most relevant determinants in the internalization of the system of roles, norms and stereotypes that form the basis of the gender socialization process.^19^

The research found that more than 50% of the adolescents expressed that the statements ‘a girl cannot get pregnant the first time she has sex’ and ‘if a girl washes herself after sex, she will not get pregnant’ were true or they did not know the answer. These two statements highlight knowledge about conception, our results being discouraging in comparison with Cordón-Colchón's research where a sample of Spanish adolescents showed lower percentages of similar sexual risk attitudes.^21^ The development of attitudes that support sexual risk activity may be a consequence of a failure in preventive care or timely sex education, whether provided by a health facility or the school they attend. Also, the usual places where adolescents seek information are often the internet or their peers, which predisposes to the dissemination of misinformation among adolescents.^22^ Another possible cause comes from within the family, given the lack of concern or parents’ rejection regarding communication on sexual health, losing the opportunity to be an important source of information for their children.^23^ Furthermore, we emphasize that more than 75% of our population belonged to the urban sector where the absence of parents due to the work day is usual.

Likewise, a higher percentage of adolescents in this study expressed disagreement about gender roles such as ‘more encouragement in a family should be given to sons than daughters to go to college’, ‘boys are better leaders than girls’, and ‘girls should be more concerned with becoming good wives and mothers than desiring a professional or business career’; similar to the results of a research study conducted at secondary schools in Ecuador and Bolivia.^17^ An analysis showed that prior to 1950, women tended to be less educated than men in all regions of the world; however, this gender gap narrowed dramatically around the world and even reversed in the advanced economies of Eastern Europe and Latin America in 2010.^24^

The research suggests that adolescents with more traditional attitudes were 1.33 times more likely to affirm or doubt ‘if a girl washes herself after sex, she will not get pregnant’. This variable could represent the lack of knowledge about a woman's fertility, which could be guided by myths or rumors and consequently produce information gaps. In the study by Markham et al. conducted in nine US schools, they found that students who ensured that ‘douching removes secretions after sex’ were 2.62 times more likely to use frequent douching to avoid pregnancy.^25^ It can be understood that these erroneous beliefs become important when adolescents try to protect themselves during sexual intercourse, choose to believe the rumors they hear around them and expose themselves to risky consequences for their health.^26^

The adjusted model showed that adolescents with more traditional attitudes were more likely to express sexual risk attitudes regarding pregnancy and STDs. Notably, 97% of participants with these traditional attitudes attended school, suggesting a potential inadequacy in sex education. This finding aligns with prior research indicating that less than 40% of Peruvians in secondary schools learned about contraception, unwanted pregnancies and prevention of HIV/STDs.^27^ Thus, sexual and reproductive health education for adolescents seems to be ineffective in Peru; it would be useful to increase the quality of knowledge about STDs and contraceptives such as condoms in schools to reduce the risk of unwanted pregnancies and venereal diseases.^28^

In contrast, this study revealed an association between showing more traditional attitudes and the probability of having sex while drunk; similar data were found in the Dominican Republic where men with a higher score on the gender role conflict scale were 56% more likely to consume alcohol during the last sexual intercourse.^29^ In recent years, the age of onset of alcohol consumption has become earlier due to predisposing factors such as family environment and social pressure; being adolescentes a more susceptible population, especially men may believe that they demonstrate their masculinity by consuming alcohol before having sex.^30^ Furthermore, the research by Ortiz-Melgar et al. revealed a significant association between alcohol consumption and sexual intercourse in Peruvian adolescents.^31^

This study has several limitations. First, the cross-sectional design prevents us from determining causality. Second, we did not include some variables that could modify these associations, such as sex education at school and unhealthy habits (alcoholism, smoking). Therefore, it is suggested that these variables be included in future studies, since they may be key points in the development of sexual risk attitudes and behaviors. Third, the results of some variables could be affected by false or insecure answers from adolescents, due to many reasons, such as embarrassment when talking about their sexual life or fear of being judged by ideologies that are not in line with the majority of their social circle. Fourth, the number of sexually active adolescents was significantly lower in relation to the total sample, so the results in this group could be biased. Nevertheless, strengths of the study include the use of a nationally representative sample and the assessment of several potential confounders. In addition, the use of cutoff points of the AWSA instrument score based on recommendations from its original validation improved this analysis. For the above reasons, the authors consider these results useful from a Peruvian and global perspective to continue the study of sexism as part of a problem and its implications for adolescent sexual and reproductive health.

### Conclusions

The results suggest an association between the expression of more traditional attitudes toward women and sexual risk attitudes. However, there was no association with most of the sexual risk behaviors studied, except for the higher probability of having at least one sexual intercourse while drunk. Although there was a higher percentage of adolescents who showed flexibility in relation to gender stereotypes, particularly men showed more traditional attitudes compared to women. This could result in a higher future risk of developing sexual risk behaviors and attitudes. In this context, it is recommended to implement comprehensive educational interventions. This can be achieved by introducing a sexual education curriculum that integrates strategies to challenge gender stereotypes, fostering both equality and respect. Also, including information about their link with sexual risk behaviors, and actively engaging parents to expand these conversations into the home environment. Finally, the continuity of research in this area is important, where longitudinal studies could provide a deeper understanding of the evolution of these attitudes and behaviors over time.

## Data Availability

The dataset is freely available for download at https://www.younglives.org.uk/.

## References

[1] Office of the United Nations High Commissioner for Human Rights. Gender stereotyping. 2020.

[2] Garcia-Vega E, Robledo EM, Izquierdo MC et al. Sexualidad, anticoncepción y conducta sexual de riesgo en adolescentes. Int J Psychol Res. 2012;5:79–87.

[3] Rangel-Flores YY, García-Rangel M. Influencia del rol de género en la conducta sexual de riesgo en adolescentes universitarios. Index Enferm. 2010;19:245–8.

[4] Organización Mundial de la Salud . La salud sexual y reproductiva del joven y del adolescente: Oportunidades, enfoques y opciones. 2009.

[5] World Health Organization . Women's health. Geneva: World Health Organization; 2018.

[6] Silver EJ, Bauman LJ. Association of “Macho Man” sexual attitudes and behavioral risks in urban adolescents. Am J Sex Educ. 2014;9:176–87.

[7] Ramiro-Sánchez T, Ramiro MT, Bermúdez MP et al. Sexism and sexual risk behavior in adolescents: Gender differences. Int J Clin Health Psychol. 2018;18:245–53. 30487930 10.1016/j.ijchp.2018.04.002 PMC6224861

[8] Shai NJ, Jewkes R, Nduna M et al. Masculinities and condom use patterns among young rural South Africa men: A cross-sectional baseline survey. BMC Public Health. 2012;12:462. 22892159 10.1186/1471-2458-12-462 PMC3504511

[9] Perrotte JK, Bibriescas N, Wainwright K et al. A bidimensional examination of machismo in relation to risky sexual cognitions and behavior among Latino college men. J Am Coll Health. 2018;68:115–8. 31305219 10.1080/07448481.2018.1538152 PMC6640084

[10] Jewkes R, Morrell R. Sexuality and the limits of agency among South African teenage women: Theorising femininities and their connections to HIV risk practices. Soc Sci Med. 2012;74:1729–37. 21696874 10.1016/j.socscimed.2011.05.020 PMC3217103

[11] Rangel-Díaz D, González-Reyes E, Barrera-Hernández M et al. Embarazo en la adolescencia: Su comportamiento en San Luis. Rev Ciencias Médicas. 2012;16:74–83.

[12] Instituto Nacional de Estadística e Informática . Las adolescentes y sus comportamientos reproductivos. 2015.

[13] Figueroa LA, Pérez LF. Conductas sexuales de riesgo en adolescentes desde el contexto cubano. Rev Ciencias Médicas. 2017;21:143–51.

[14] Sánchez A . Diseño y métodos del estudio Niños del Milenio—Perú. GRADE, Grupo de Análisis para el Desarrollo; 2018. Available (Peru): https://www.ninosdelmilenio.org/wp-content/uploads/2018/03/NI%C3%91OS-DEL-MILENIO_5.pdf.

[15] Von Elm E, Altman DG, Egger M et al. The strengthening the reporting of Observational Studies in Epidemiology (STROBE) statement: Guidelines for reporting observational studies. J Clin Epidemiol. 2008;61:344–9. 18313558 10.1016/j.jclinepi.2007.11.008

[16] Jaruseviciene L, De Meyer S, Decat P et al. Factorial validation of the Attitudes toward Women Scale for adolescents (AWSA) in assessing sexual behaviour patterns in Bolivian and Ecuadorian adolescents. Glob Health Action. 2014;7:23126. 24461355 10.3402/gha.v7.23126 PMC3901847

[17] De Meyer S, Jaruseviciene L, Zaborskis A et al. A cross-sectional study on attitudes toward gender equality, sexual behavior, positive sexual experiences, and communication about sex among sexually active and non-sexually active adolescents in Bolivia and Ecuador. Glob Health Action. 2014;7:24089. 25024066 10.3402/gha.v7.24089 PMC4095758

[18] Kemigisha E, Nyakato VN, Bruce K et al. Adolescents’ sexual wellbeing in southwestern Uganda: A cross-sectional assessment of body image, self-esteem and gender equitable norms. Int J Environ Res Public Health. 2018;15:372. 29470388 10.3390/ijerph15020372 PMC5858441

[19] García-Viamontes D, Carbonell-Vargas M. Los estereotipos de género: Un estudio en adolescentes. Estudios Desarrollo Social. 2023;11:e14.

[20] World Economic Forum . Global Gender Gap Report 2023. 2023. https://www.weforum.org/publications/global-gender-gap-report-2023/ [accessed 25 November 2023].

[21] Cordón-Colchón J . Mitos y creencias sexuales de una población adolescente de Almendralejo. Matronas prof. 2008;9:6–12.

[22] Hust SJT, Brown JD, L'Engle KL. Boys will be boys and girls better be prepared: An analysis of the rare sexual health messages in young adolescents’ media. Mass Commun Soc. 2008;11:3–23.

[23] Eversole JS, Berglas NF, Deardorff J et al. Source of sex information and condom use intention among Latino adolescents. Health Educ Behav. 2017;44:439–47. 27899688 10.1177/1090198116671704

[24] Permanyer I, Boertien D. A century of change in global education variability and gender differences in education. PLoS One. 2019;14:e0212692. 30811455 10.1371/journal.pone.0212692 PMC6392467

[25] Markham CM, Tortolero SR, Addy RC et al. Factors associated with frequent vaginal douching among alternative school youth. J Adolesc Health. 2007;41:509–12. 17950172 10.1016/j.jadohealth.2007.05.022 PMC2083649

[26] Álvarez-Muelas A, Gómez-Berrocal C, Sierra JC. Relación del doble estándar sexual con el funcionamiento sexual y las conductas sexuales de riesgo: Revisión sistemática. Rev iberoam psicol salud. 2020;11:103–16.

[27] Motta A, Keogh S, Prada E et al. De la normativa a la práctica: La política de educación sexual y su implementación en el Perú. Guttmacher Institute; 2017. Available (USA): https://www.guttmacher.org/es/report/ politica-de-educacion-sexual-peru.

[28] Pérez LL, Jenaro C, Rodríguez-Becerra M et al. Los roles de género y su papel en las actitudes y comportamientos afectivo-sexuales: Un estudio sobre adolescentes salmantinos. Cuest género. 2016;16:457–76.

[29] Fleming PJ, Barrington C, Powell W et al. The association between men's concern about demonstrating masculine characteristics and their sexual risk behaviors: Findings from the Dominican Republic. Arch Sex Behav. 2018;47:507–15. 27844313 10.1007/s10508-016-0880-6 PMC5429985

[30] Delgado J, Bravo M, Palos P. Consumo de alcohol y conducta sexual de riesgo en adolescentes. Psychol Int. 2007;18:1–13.

[31] Ortiz- Melgar M, Pérez-Saavedra V, Valentín-Ballarta JJ et al. Asociación entre consumo de alcohol y relaciones sexuales ocasionales en los adolescentes. Rev enferm Herediana. 2015;8:109–14.

